# Population of invasive group A streptococci isolates from a German tertiary care center is dominated by the hypertoxigenic virulent M1_UK_ genotype

**DOI:** 10.1007/s15010-023-02137-1

**Published:** 2023-12-08

**Authors:** Manuel Wolters, Benjamin Berinson, Nicole Degel-Brossmann, Armin Hoffmann, Rico Bluszis, Martin Aepfelbacher, Holger Rohde, Martin Christner

**Affiliations:** Institut für Medizinische Mikrobiologie, Virologie und Hygiene, Universitätsklinikum Hamburg-Eppendorf (UKE), Martinistrasse 52, 20246 Hamburg, Germany

**Keywords:** *Streptococcus pyogenes*, Epidemiology, *Emm* type, M1_UK_

## Abstract

**Purpose:**

Hypertoxigenic *Streptococcus pyogenes emm*1 lineage M1_UK_ has recently been associated with upsurges of invasive infections and scarlet fever in several countries, but whole-genome sequencing surveillance data of lineages circulating in Germany is lacking. In this study, we investigated recent iGAS isolates from our laboratory at a German tertiary care center for the presence of the M1_UK_ lineage.

**Methods:**

Whole-genome sequencing was employed to characterize a collection of 47 consecutive non-copy isolates recovered from blood cultures (21) and tissue samples (26) in our laboratory between October 2022 and April 2023.

**Results:**

M protein gene (*emm*) typing distinguished 14 different *emm* types, with *emm*1 (17) being the dominant type. Single-nucleotide polymorphism (SNP) analysis confirmed the presence of all 27 SNPs characteristic for the M1_UK_ lineage in 14 of 17 *emm*1 isolates.

**Conclusion:**

This study has shown for the first time that M1_UK_ is present in Germany and might constitute a driving force in the observed surge of GAS infections. This observation mirrors developments in the UK and other countries and underscores the importance of WGS surveillance to understand the epidemiology of GAS.

**Supplementary Information:**

The online version contains supplementary material available at 10.1007/s15010-023-02137-1.

## Introduction

**Fig. 1:**
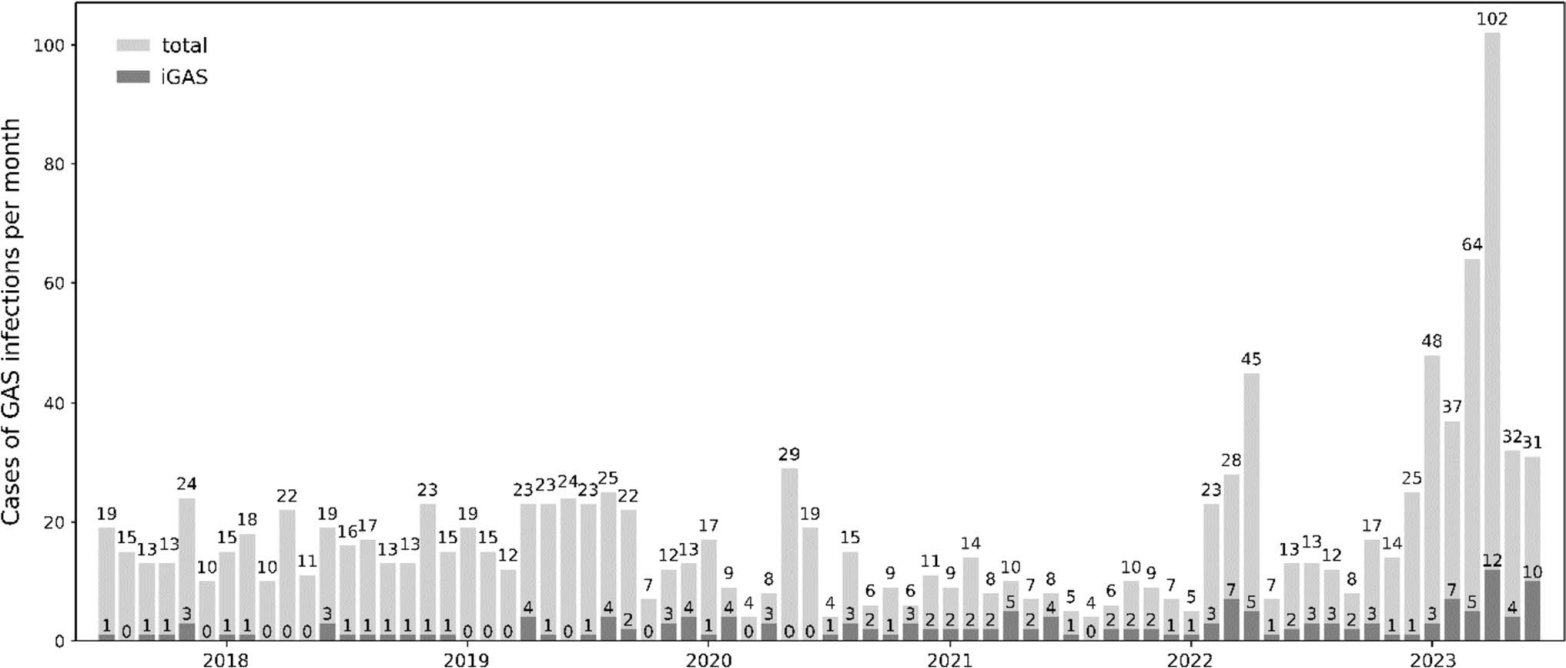
Monthly cases of GAS infections identified by our laboratory between 7/2017 and 6/2023. Total number of cases and cases of invasive infections (iGAS) are represented by light and dark grey bars, respectively. Numbers above the light grey bars indicate the number of all GAS detections in the respective period, and numbers above the dark grey bars indicate the number of iGAS

*Streptococcus pyogenes*, also referred to as Group A *Streptococcus* (GAS), is an important human pathogen that causes non-invasive infections such as scarlet fever, pharyngitis and impetigo, and also life-threatening invasive infections (iGAS) such as necrotising fasciitis, pneumonia, meningitis and puerperal sepsis [1]. In 2022 and 2023, several European countries (including Denmark, Ireland, France, the Netherlands, Sweden, Spain, and the UK) have reported a marked increase in scarlet fever and iGAS [2–6]. The observed increase followed a period of low incidence during the COVID-19 pandemic, but has now exceeded pre-pandemic levels [5, 6]. This phenomenon is likely attributable to reduced exposure at the population level and an associated so-called immunity gap [7], which may have led to widespread dissemination in the population after the lifting of COVID-19-related restrictions. In addition, the current high activity of viral respiratory infections might have contributed to an increase in iGAS cases with a respiratory focus [6, 8]. On the other hand, an increase of scarlet fever and iGAS had been observed in the UK some years before the pandemic and was associated with the emergence and spread of a new lineage of *S. pyogenes* designated M1_UK_ [9]. The M1_UK_ lineage is a variant of the highly successful, contemporary epidemic M1_global_ strain [10]. M1_UK_ is differentiated from M1_global_ by 27 chromosomal single nucleotide polymorphisms (SNPs) and exhibits enhanced expression of the superantigenic scarlet fever toxin SpeA (streptococcal pyrogenic exotoxin A) in vitro [9, 11]. The M1_UK_ lineage has subsequently been identified in several other countries (Australia, Belgium, Canada, Netherlands, Portugal, Scotland, USA) [3, 8, 11–15], where in some cases (Australia, Belgium, Netherlands, Portugal) it has also expanded and displaced the M1_global_ lineage [11, 12, 16, 17]. Recently, the emergence and spread of two more new clones designated M1_DK_ (*emm1*) and M4_NL22_ (*emm4*) were reported in Denmark and the Netherlands, respectively [4, 18]. In Germany, the Robert Koch Institute (RKI) also reported a strong increase in iGAS infections for the fourth quarter of 2022 [19]. RKI surveillance data also show a marked increase in the number of reported scarlet fever cases from two federated German states with mandatory scarlet fever reporting (https://survstat.rki.de/; accessed 2023/08/25). This trend was reflected in the number of GAS/iGAS isolates recovered from clinical samples at our laboratory (Fig. 1). Currently, whole-genome surveillance data of circulating *S. pyogenes* strains from Germany is not yet available. To explore the GAS population and to elucidate whether any of the epidemic clones recently described in neighbouring countries might have contributed to the reported increase in iGAS infections, we analysed a collection of 47 *S. pyogenes* isolates recovered from blood and tissue samples from October 2022 to April 2023 by whole-genome sequencing.

## Results and discussion

**Fig. 2:**
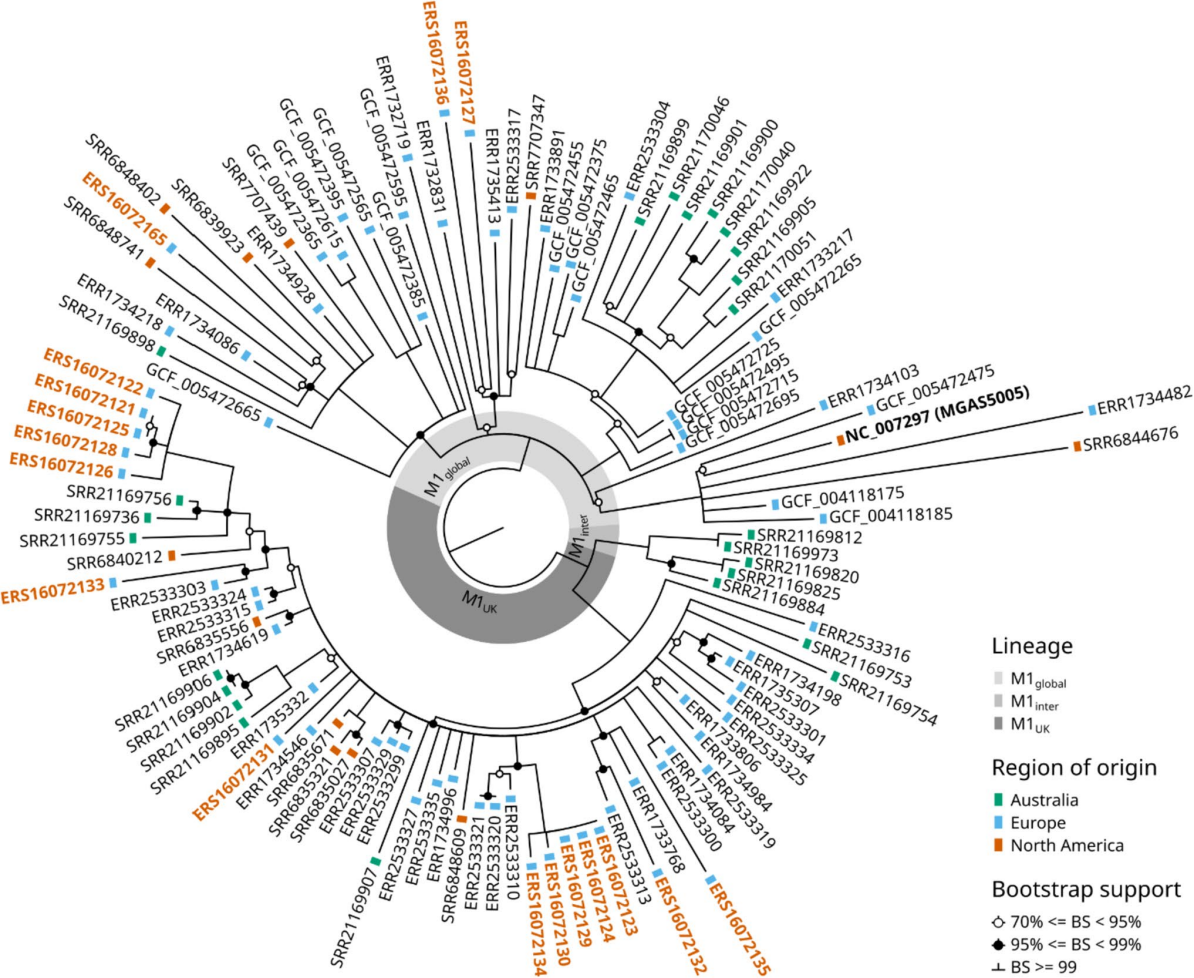
Maximum likelihood phylogenetic tree from core SNPs (excluding prophage regions). Isolates are labelled with accession numbers; isolates sequenced for the present study are shown in orange boldface print. M1 lineage is indicated by shading of the inner doughnut plot. Region of strain origin is indicated by coloured rectangles. Nodes with bootstrap support values lower than 70% have been collapsed, and bootstrap support values between 70 and 99% are marked with circles

The *S. pyogenes* M1_UK_ lineage has emerged and rapidly spread in several countries worldwide. Owing to the lack of nationwide whole-genome surveillance data for *S. pyogenes*, information on the presence of the M1_UK_ lineage in Germany is not yet available. This prompted us to characterize the population structure of contemporary *S. pyogenes* isolated during routine diagnostic workup of blood cultures and tissue samples at the microbiology laboratory of the University Medical Center Hamburg-Eppendorf, a 1600-bed tertiary care center. Between October 2022 and April 2023, a total of 53 non-copy *S. pyogenes* isolates were recovered from eligible specimens. Of those, 47 (21 blood culture isolates and 26 isolates from tissue specimens) were available for whole-genome sequencing and subsequent delineation of the recently described new M1_UK,_ M1_DK_ and M4_NL22_ lineages (supplemental file 1). M protein gene (e*mm)* typing distinguished 14 different *emm* types, with *emm*1 [17] being the dominant type, followed by *emm*89 [7] (Table 1). Single-nucleotide polymorphism (SNP) analysis confirmed the presence of all 27 SNPs characteristic for the M1_UK_ lineage [9] in 14 of 17 *emm*1 isolates. The remaining three *emm*1 isolates lacked any of the M1_UK_-defining SNPs. SNP-based phylogenetic analysis grouped our M1_UK_ isolates together with representative M1_UK_ isolates recovered from iGAS in the UK, Australia, Canada and the USA and separated them from the M1_global_ and M1_inter_ lineages. M1_inter_ lineages carry subsets of the SNPs that define M1_UK_, but failed to spread as successful as M1_UK_ [9, 11] (Fig. 2). Analysis of virulence and resistance gene content of our 14 M1_UK_ isolates revealed no striking differences as compared to other M1_UK_ or local M1_global_ strains (supplemental file 1) [9]. The *emm*1 clone M1_DK_, reported to be highly prevalent in Denmark [4], was not found, and only one isolate of *emm*4, a prevalent genotype in invasive infections in the Netherlands [18], was identified in our collection.

**Table 1:** Distribution of *emm* types

*emm* type	Number [*n*]
1	17 (14 M1_UK_)
12	6
89	6
27	3
49	3
53	2
66	2
87	2
4	1
11	1
77	1
102	1
106	1
218	1
Total	47

In conclusion, this study has shown for the first time that M1_UK_ is present in Germany and might constitute a driving force in the observed surge of GAS infections. We concede that our study is only a snapshot of a regional *S. pyogenes* population, which may not be representative of the German *S. pyogenes* population. In addition, due to the lack of patient travel information, acquisition of M1_UK_ GAS outside of Germany cannot be excluded in all cases. Our study population also did not encompass commensal isolates or isolates from cases of uncomplicated pharyngitis and might thus not reflect the overall composition of our local *S. pyogenes* population. Nevertheless, our preliminary data underscore the need for further studies of larger strain collections to reconstruct the spread of M1_UK_ in Germany and elucidate its role in the current surge of GAS infections [19].

## Methods

*S. pyogenes* study isolates (n = 47) were thawed from a − 80 °C cryo-collection of contemporary isolates and underwent WGS. In brief, DNA was extracted using QiaSymphony mericon extraction kits (Qiagen) on a QiaSymphony SP instrument according to the manufacturer’s instructions. Libraries for paired end sequencing were prepared using the NEB NextUltra II DNA library Prep Kit for Illumina (NEB) and sequenced with 2 × 150 cycles on an Illumina MiSeq instrument. Reads or nucleotide sequences from additional isolates were obtained from the National Center for Biotechnology Information (NCBI) sequence read archive, the NCBI reference sequence database and the European Nucleotide Archive (ENA). Reads were assembled with shovill 1.1 employing spades 3.15.5 [20], annotated with bakta 1.8.1 [21] and subjected to pan genome analysis with roary 3.13.0 [21]. *Emm* types were assigned from bakta assemblies using the Centers for Disease Control *Streptococcus* Laboratory GAS bioinformatic pipeline (https://github.com/BenJamesMetcalf/GAS_Scripts_Reference). Resistance genes where detected using AMRFinderPlus 3.11.14 with the NCBI reference gene database version 2023–08-08.2 [22]. Known virulence markers were identified with abricate 1.0.1 (https://github.com/tseemann/abricate) and the virulence factor database version 2022–08-26 [23]. Additional allelic profiling was performed with chewBBACA 3.3.0 [24] and a *S. pyogenes* wgMLST schema from Chewie-NS [25]. Core SNPs were identified with snippy 4.6.0 (https://github.com/tseemann/snippy) using *S. pyogenes* MGAS5005 (GenBank accession NC_007297.2) as a reference. Maximum likelihood phylogenies from concatenated core SNPs were constructed using gubbins 3.3.0 [26] with IQTree [27] and visualized with TreeViewer 2.1.0 (https://github.com/arklumpus/TreeViewer/tree/v2.1.0).

### Supplementary Information

Below is the link to the electronic supplementary material.

Supplementary file1 (ODS 36 kb)

## Data Availability

Whole genome sequences generated for this study are available in the European Nucleotide Archive at https://www.ebi.ac.uk/ena/browser/home and can be accessed with the project number PRJEB64404. Isolate metadata are available in the online supplementary material.
